# Gestational psittacosis in a Montana sheep rancher.

**DOI:** 10.3201/eid0302.970214

**Published:** 1997

**Authors:** D M Jorgensen

## Abstract

In humans, psittacosis is primarily a flulike illness following exposure to psittacine birds. In rare cases, pregnant women exposed to Chlamydia psittaci can contract gestational psittacosis: atypical pneumonia, sepsis, and placental insufficiency resulting in premature birth or miscarriage. In the United States, only two cases of gestational psittacosis have been reported, both from exposure to psittacine birds. Eleven other cases have been reported worldwide, mostly in the United Kingdom, all from exposure to infected birth fluids and membranes of farm mammals, notably sheep and goats. In these mammals, C. psittaci inhabit the reproductive tract, are transmitted sexually or by the fecal-oral route, and cause miscarriages. The case of gestational psittacosis in a Montana sheep rancher is the first farm animal-related case reported in the United States. Pregnant women should avoid close contact with C. psittaci-infected animals, particularly sheep and goats during the birthing season. Obstetricians should consider this diagnosis along with early antibiotic treatment and cesarean section delivery in the context of the patient's case history.


### Gestational Psittacosis in a Montana Sheep Rancher

In humans, psittacosis is primarily a flulike illness following exposure to psittacine birds. In rare cases, pregnant women exposed to *Chlamydia psittaci* can contract gestational psittacosis: atypical pneumonia, sepsis, and placental insufficiency resulting in premature birth or miscarriage. In the United States, only two cases of gestational psittacosis have been reported, both from exposure to psittacine birds. Eleven other cases have been reported worldwide, mostly in the United Kingdom, all from exposure to infected birth fluids and membranes of farm mammals, notably sheep and goats. In these mammals, *C. psittaci* inhabit the reproductive tract, are transmitted sexually or by the fecal-oral route, and cause miscarriages. The case of gestational psittacosis in a Montana sheep rancher is the first farm animal–related case reported in the United States. Pregnant women should avoid close contact with *C. psittaci*–infected animals, particularly sheep and goats during the birthing season. Obstetricians should consider this diagnosis along with early antibiotic treatment and cesarean section delivery in the context of the patient's case history.

Psittacosis is a flulike systemic infection, often with fever, headache, and atypical pneumonia, caused by *Chlamydia psittaci*. The number of reported cases varies from year to year because of periodic outbreaks, although the true baseline incidence in the United States is thought to be 75 to 100 cases per year with 1 or 2 deaths (1). Montana typically reports one case per year (Montana Dept. of Health surveillance data). Many cases are probably not diagnosed. In most reported cases, transmission occurs by inhaling infectious material from diseased psittacine birds (2).

However, not all cases of psittacosis result from inhaling avian strains of *C. psittaci*, nor are they all manifested as a simple flulike illness or atypical pneumonia. In rare cases, pregnant women contract severe chlamydial disease after exposure to the infected birth fluids and membranes of goats and sheep (3). This "gestational psittacosis" can be defined as a flulike syndrome leading to sepsis, placental infection, and fetal compromise (4). Only 13 cases have been reported worldwide: Eleven after exposure to gravid sheep and goats (United Kingdom and France) and two after exposure to psittacine birds (United States). This is the first farm animal–related case reported in the United States.

### Case Report

In April 1996, a previously healthy 25-year-old Montana sheep rancher, pregnant for the first time, had cough and congestion for 14 days at weeks 19 to 21 of gestation; the symptoms were followed by 4 days of high fever (104°F), myalgia, headache, fatigue, and backache, and 2 days of abdominal pain. Upon hospitalization, a chest X-ray was normal, but laboratory tests showed anemia (Hg 10.7 g/dl), thrombocytopenia (platelets 42,000/µl), and hepatic dysfunction (SGOT 351 IU/L), as well as proteinuria (2+) and hematuria (1+). Other tests showed a positive lupus anticoagulant and positive anticardiolipin antibodies. Blood, urine, and sputum cultures (collected before antibiotics were administered) yielded negative results. Admitting diagnosis was "early severe and low platelet count) syndrome" with a possible concurrent viral infection and poor hydration accounting for the fever and lack of hypertension.

On the second day of hospitalization, the patient underwent emergency termination of the pregnancy (22-23 weeks gestation). On the following day, the patient still had high fevers; moderate respiratory distress with tachypnea developed, and oxygen was required. A chest X-ray showed diffuse bilateral alveolar infiltrates; antibiotic treatment (IV Ancef) was started for presumed pneumonia. The fever subsided, the patient's condition improved remarkably, and she was discharged within 3 days. The discharge diagnosis was early severe HELLP syndrome (probably related to lupus anticoagulant and anticardiolipin antibodies) with coincident pneumonia. On follow-up, the patient was feeling well and had resumed her activities around the ranch.

Suspicious of a diagnosis related to environmental exposure and motivated by a need to explain all of the patient's symptoms, the obstetrician sent placental and fetal tissue to California and Oklahoma for specialized pathologic examination, after which patient sera were sent to the Centers for Disease Control and Prevention (CDC) in Atlanta, Georgia, and animal sera were sent to the Veterinary Diagnostic Laboratory in Bozeman, Montana. A concomitant epidemiologic investigation was initiated.

Hematoxylin and eosin stain of the placenta showed placentitis: an intense acute inflammation of the intervillous space, more on the maternal side, which suggested infection from hematogenous spread rather than from ascension from the birth canal. Numerous basophilic intracytoplasmic inclusions in the cytoplasm of the trophoblast appeared to contain minute cocci-shaped organisms. The inclusions were identified by genus-specific fluorescein-tagged monoclonal antibody staining as masses of *Chlamydia* (either *pneumoniae* or *psittaci*) in various stages of development. Electronmicrographs further defined the round, rather than pear-shaped, morphologic features of the elementary bodies, which suggested *psittaci* rather than *pneumoniae* as the specific species. The pathologic findings are reviewed in greater detail, including photographs, in a concurrent publication (4).

Because traditional complement-fixation serologic tests do not adequately differentiate the chlamydial species in humans, sera were sent to CDC for a microimmunofluorescence assay, which is more sensitive and specific (5). The patient's sera had very high (1:512) IgG antibody titer to *C. psittaci*; identical titer was seen on sera collected several months later. No IgM to *C.psittaci* was detected. Tests for the other chlamydia, as well as for other placental-acquired infections (e.g., Q fever and brucellosis) were negative.

The epidemiologic investigation showed that, during the week before hospitalization, the patient's husband had a similar flulike illness with mild respiratory symptoms. The spring lambing season began 4 to 6 weeks before hospitalization, during which the patient and her husband worked closely with gravid sheep.

Out of a flock of 24 sheep, 19 gave birth to 30 lambs. There were six lamb deaths but no reported abortions. The patient assisted in most deliveries; however, she had direct contact on just three occasions: pulling one lamb during a twin birth, pulling one kid during the twin birth of her pet goat, and assisting in the premature delivery of a yearling ewe. The latter resulted in considerable placental exposure. She spent approximately 1 hour manually extracting the retained placenta, wearing only plastic gloves; she did not cover the mouth or nose. Serologic testing of five sheep and two goats was performed by complement fixation for *C. psittaci*. Only sera from two sheep were positive, including the yearling ewe from which the patient manually extracted the retained placenta.

The sheep were purchased from a neighboring flock with reported "pink eye" and abortions, both of presumed chlamydial etiology. The flock's abortion problem resolved after tetracycline-fortified feed was administered just before yearly breeding. Neither antibiotic feed nor the available chlamydia vaccine was used for the sheep on the patient's ranch. During her pregnancy, the patient had minimal contact with other ranch animals, including cattle, horses, and poultry; none of these animals were ill or had abortions. She had no contact with psittacine birds.

HELLP is a variant of the preeclampsia-eclampsia syndrome, which also includes epigastric pain and liver tenderness, as well as hypertension, edema, and proteinuria. An under-appreciated early symptom is generalized malaise and/or flulike illness (6,7). Antiphospholipid antibodies, such as the lupus anticoagulant and anticardiolipin antibodies, have been associated with thrombosis, thrombocytopenia, and pregnancy complications, particularly decidual vasculopathy, placental infarction, fetal growth retardation, early-onset preeclampsia, recurrent miscarriages, and fetal death (8). Initially, in this patient, except for the high fever and lack of hypertension, the symptoms reasonably conformed to those of HELLP syndrome. However, in this case, epidemiologic, clinical, laboratory (human and animal), and histopathologic data support the diagnosis of gestational psittacosis. The most likely mechanism of transmission was inhalation of *C.psittaci* while manually extracting the retained placenta from the *C. psittaci*–infected sheep that lambed prematurely just 2 weeks before the patient's hospitalization. The usual incubation time for psittacosis is 1 to 4 weeks (2).

In the United States, pneumonia acquired through contact with birth membranes or fluids of farm animals is more commonly caused by *Coxiella burnetti* (Q-fever) or *Brucella sp*.

(brucellosis) (9). In both diseases, the flulike syndrome does not lead to fetal compromise in pregnant women. However, in gestational psittacosis, the flulike illness results in sepsis, placental infection, and fetal compromise. The disease is relatively new, first reported in the United Kingdom in 1967, and rare, just 13 cases reported worldwide (4,10,11). Gestational psittacosis has occurred from exposure to psittacine birds (the United States: two previous cases; 12,13) and from exposure to infected birth fluids and membranes of sheep or goats (the United Kingdom: nine cases, all sheep [10,11,14-19]; France: two cases, both goats [20,21]). This is, therefore, the third case reported in the United States, the first related to farm animals (4).

Further review of the clinical details of the 13 other reported cases shows remarkable similarity between them and the case reported here. For example, in all 14 reported cases (including this case) the illness is manifested as a flulike febrile syndrome. Similarly, in the 14 cases, thrombocytopenia and/or coagulopathy were reported (12 [85%]), as well as pulmonary disease (12 [85%]) and liver disease (11 [78%]). The outcome of pregnancy is also similar: 11 (78.5%) of 14 pregnancies ended in fetal death, while the three surviving neonates were born by cesarean section before 34 weeks gestation (4). Maternal outcome was much better: 13 of the 14 women recovered fully after pregnancy termination. The other woman died of overwhelming sepsis. Erythromycin was administered to six of the 14 pregnant women, including the mothers of the three surviving infants, early in the illness (4,10,12,13). Tetracycline, the drug of choice for psittacosis, is usually contraindicated during pregnancy, although it is still recommended for cases of gestational psittacosis that appear refractory to erythromycin (12,13).

*C. psittaci* is known worldwide as the primary cause of abortion in sheep, often referred to as “enzootic abortion of ewes”, “chlamydial abortion,” or simply “enzootic abortion.” Enzootic abortion in sheep was first reported in Scotland in 1936, where *C. psittaci* became known as the “ewe abortion agent” (10,22). Abortion usually occurs within 2 years of contact with other aborting sheep in the flock. Indeed, pregnant sheep experimentally infected with an ovine abortion isolate of *C.psittaci* either abort or give birth to weak lambs and maintain a persistent systemic antibody response to the organism for up to 2.5 years postinfection; for sheep that aborted, detectable amounts of chlamydial antigen are excreted from the reproductive tract during subsequent estrus cycles (23). Postpartum (or postabortive) infected sheep are considered protected from subsequent *C. psittaci*–induced pregnancy complications, and they remain healthy, fertile, and therefore able to transmit the organism within the flock, perhaps through sexual contact (23,24).

Moreover, *C. psittaci* organisms are found in eye, respiratory, oral, and fecal material, which suggests other modes of transmission. Indeed, environmental contamination from diseased placentae or vaginal discharge is considered the primary source of infection to other sheep, perhaps through ingestion of infected tissue or contaminated feed during the previous or current lambing season and subsequent intestinal colonization (23,24). The carrier state is not evident but is often presumed if a high incidence of abortions or premature lambing occurs in the flock. Sometimes a high rate of “pink eye” in the flock indicates high *C. psittaci* prevalence, although this is usually caused by a serovar different from the one causing abortion. Nevertheless, ranchers who suspect *C.psittaci* in the flock typically respond by physically isolating infected sheep, giving antibiotic feed, and/or using the chlamydial vaccine. However, these methods rarely eliminate the disease from the flock (23).

In Montana, 50 to 100 aborted sheep fetuses are examined each year, more than 50% of which test positive for *C. psittaci* by complement fixation on sera or enzyme-linked immunosorbent assay on placental tissue. For this reason, masks are always worn by laboratory workers during sheep (and psittacine bird) autopsies (L. Stackhouse, Montana Veterinary Diagnostic Laboratory, pers. comm.). It is difficult to estimate the overall rate of colonization/infection within sheep flocks, although it is presumed to be high, particularly in the United Kingdom, and in areas of the United States where sheep abortions have already occurred and feed-antibiotics or chlamydial vaccine is not routinely used. High rates of sheep infection, thus far, have not led to high rates of miscarriage in female sheep ranchers. Nevertheless, given the increasing number of small family farms or hobby farms in the western United States, a growing number of farmers could be raising sheep (and other animals) without knowing the potential hazards to human health and how to prevent them.

Thus, it is strongly recommended that pregnant women avoid contact with membranes or birth fluids of sheep and goats and close contact with psittacine birds during pregnancy. Moreover, obstetricians should consider the diagnosis of gestational psittacosis in pregnant patients initially presumed to have HELLP syndrome and/or a flulike illness, particularly during the spring lambing season, so that appropriate early antibiotics and cesarean section delivery can help reduce illness and death from the disease.

### Acknowledgments

The author would like to thank Drs. Mark Miles (Great Falls, MT), Mary Ballinger (Belt, MT), Larry Stackhouse (Bozeman, MT), Kurt Benirschke (San Diego, CA), Scott Hyde (Tulsa, OK), and K.H. Wong (Atlanta, GA) for their technical contributions to this investigation and their encouragement and expert advice.

**Daniel M. Jorgensen**
Montana State Medical Officer

### References

1. Centers for Disease Control and Prevention. Summary of Notifiable Diseases, 1995. MMWR Morb Mortal Rep 1995;44(53 suppl):50-74.
2. Benenson A, editor. Control of communicable diseases manual, 16th edition. American Public Health Association, 1995;377-9.
3. Sclossberg D. *Chlamydia psittaci*. In: Mandel G, editor. Principles and practice of infectious diseases, 4th edition, 1995. New York: Churchill Livingstone, Inc.;1694-5.
4. Hyde SR. Gestational psittacosis: report of a case with literature review. Mod Path. In press.
5. Wong KH, Skelton SK, Daugherty H. Utility of complement fixation and microimmunofluourescence assays for detecting serologic responses in patients with clinically diagnosed psittacosis. J Clin Microbiol 1994;32:2417-21.
6. Cunningham F, MacDonald P, Gant N, Levene K, Gilstrap L, editors. Hypertensive disorders in pregnancy. In: Williams Textbook of Obstetrics. Norwalk (CT): Appleton and Lange, 1994; 763-90.
7. Tomsen TR. HELLP syndrome (hemolysis, elevated liver enzymes, and low platelets) presenting as generalized malaise. Am J Obstet Gynecol 1995;172:1876-80.
8. Cunningham F, MacDonald P, Gant N, Levene K, Gilstrap L, editors. Connective tissue disorders. In: Williams Textbook of Obstetrics. Norwalk (CT): Appleton and Lange, 1994; 1229-37.
9. Donowitz G, Mandell G. In: Mandel G, editor. Acute Pneumonia. In: Principles and practice of infectious diseases. 4th edition.New York: Churchill Livingstone, Inc., 1995;619-34.
10. Beer RJS, Bradford WP, Hart RJC. Pregnancy complicated by psittacosis acquired from sheep. BMJ 1982;284:1156-7.
11. Roberts W, Grist NR, Giroud P. Human abortion associated with infection by ovine abortion agent. BMJ 1967;4:37.
12. Gherman RB, Leventis LL, Miller RC. Chlamydial psittacosis during pregnancy: a case report. Obstet Gynecol 1985;86:648-50.
13. Khatib R, Huthayipailayam C, Thirumoorthi M, Kelly B, Grady K. Severe psittacosis during pregnancy and suppression of antibody response with early therapy. Scand J Infect Dis 1995;27:519-21.
14. Johnson FW, Matheson BA, Williams H, Laing AG, Jandial V, Davidson-Lamb R, et al. Abortion due to infection with *Chlamydia psittaci* in a sheep farmer's wife. BMJ 1985;290:592-4.
15. Wong SY, Gray ES, Buxton D, Finlayson J, Johnson FW. Acute placentitis and spontaneous abortion caused by *Chlamydia psittac*i of sheep origin: a histological and ultrastructural study. J Clin Pathol 1985;38:707-11.
16. Helm CW, Smart GE, Cumming AD, Lambie AT, Gray JA, MacAulay A, Smith IW.. Sheep-acquired severe *Chlamydia psittaci* infection in pregnancy. Int J Gynaecol Obstet 1989;28:369-72.
17. McGivern D, White R, Paul ID, Caul EO, Roome AP, Westmoreland D. Concomitant zoonotic infections with ovine Chlamydia and Q fever in pregnancy: clinical features, diagnosis, management, and public health implications. Case report. Br J Obstet Gynaecol 1988;95:294-8.
18. Crosse BA, Gomes P, Muers MM. Ovine psittacosis and sarcoidosis in a pregnant woman. N Engl J Med 1971;284:642-53.
19. Hadley KM, Carrington D, Frew CE, Gibson AA, Hislop WS. Ovine chlamydiosis in an abattoir worker. J Infect 1992;25:105-9.
20. Villemonteix P, Agius G, Ducroz B, Rouffineau J, Plocost V, Castets M, Magnin G. Pregnancy complicated by severe *Chlamydia psittaci* infection acquired from a goat flock: a case report. Eur J Obstet Gynecol Repr Biol 1990;37:91-4.
21. Berthier M, Bonneau D, Marechaud M, Oriot D, Deshayes M, Leillain P, Magnin G. Maternal-fetal infection by *Chlamydia psittaci* transmission from a goat: a new zoonosis? Bull Soc Path Exot 1991;84:590-6.
22. Hart CA, Broadhead RL. Neonatal infections. In: A Colour Atlas of Paediatric Infectious Diseases. Aylesbury, England: Wolfe Publishing, 1992;18-9.
23. Papp JR, Shewen PE, Gartley CJ. Abortion and subsequent excretion of chlamydiae from the reproductive tract of sheep during estrus. Infect Immun 1994;62:3786-92.
24. Papp JR, Shewen PE. Localization of chronic *Chlamydia psittaci* infection in the reproductive tract of sheep. J Infect Dis 1996;174:1296-301.
